# Limitations of informed consent in routine clinical practice - An observational survey study to characterize patient populations with limited understanding of the routine informed consent process in an anaesthesia clinic

**DOI:** 10.1186/s12871-025-03275-9

**Published:** 2025-07-30

**Authors:** Elisabeth Bär, Tobias Bock, Bettina Bock, Anne Goldbach, Julia Heusner, Saskia Schuppener, Sven Bercker

**Affiliations:** 1 Faculty of Medicine, University of Leipzig, Leipzig, Germany; 2 Department of Anesthesiology and Intensive Care Medicine, University Hospital of Leipzig , Leipzig, Germany; 3 Institute for German Language and Literature II, University of Cologne , Cologne, Germany; 4 Faculty of Education, Institute of special needs and inclusive education, University of Leipzig, Leipzig, Germany

**Keywords:** Informed consent, Health literacy, Anaesthesia consultation, Preoperative education, Socioeconomic factors in healthcare, Ethical aspects of informed consent, Barriers to informed consent

## Abstract

**Background:**

Informed consent is a fundamental component of patient autonomy and legal protection. Effective understanding of medical information depends on health literacy, which is influenced by factors such as age, educational level and cognitive ability. This study aims to identify patient groups with limited understanding of the informed consent process for anaesthesia and to assess the factors influencing their understanding.

**Methods:**

A monocentric, prospective, observational survey study was conducted at the Anaesthesia Clinic of the University Hospital of Leipzig. A total of 501 patients were interviewed using a structured questionnaire, 489 were analysed for demographic data. 12 patients were excluded in the first place due to incomplete data and a further 135 could not be used in the analysis for the comparison groups. Participants were divided into two groups: All patients who answered at least one of the comprehension-related questions incorrectly were assigned to group V0 (limited understanding), while V1 (full understanding) contained all those who were able to answer all questions correctly. Comparisons between groups were conducted via Mann-Whitney U test for ordinal and continuous variables, and the chi-square (χ²) test for nominal variables.

**Results:**

Patients with limited understanding were generally older, less likely to be employed (including retired patients), and had lower educational attainment. They also had a higher need for physical assistance. A history of psychiatric treatment was more common in those with full understanding. No significant differences were observed regarding non-physical assistance, anaesthetist experience, length of consultation or the proportion of non-German speakers and foreign-born patients.

**Conclusions:**

Comprehension of medical information is influenced by individual factors and patient health literacy. Targeted communication strategies, such as the use of simplified language and visual aids, are needed to improve patient understanding. Future research should focus on the development and implementation of interventions to improve patient understanding and overall health literacy.

**Supplementary Information:**

The online version contains supplementary material available at 10.1186/s12871-025-03275-9.

## Introduction

Medical consultation regarding informed consent is crucial for enabling patients to make informed decisions about medical procedures and simultaneously serves as legal protection for physicians. Its primary aim is to empower patients to make independent, autonomous and well-informed decisions. To achieve this, patients must be able to comprehend the information presented to them and apply it to their specific circumstances. However, personal factors such as cognitive limitations or the complexity of the situation can make this process more challenging.

The process of informed consent in anaesthesia is complicated by several factors, including legal and communicative issues, time pressure, standardised procedures and communication problems. The relationship between anaesthetist and patient, as well as structural conditions in everyday clinical practice, pose significant challenges [1]. 

Patients often retain only parts of their conversations, leading to misunderstandings or poor recall. This has already become apparent in previous studies. In Gillies et al., patients were asked about anaesthetic complications two weeks after being informed on the day of surgery. A quarter could not remember any of the complications, and 37% could not remember the major risks either [2]. Parents who have been informed about the risks associated with their children’s operations find themselves in a comparable situation, with only half of them able to recall the risks [3]. 

Another concept for approaching this problem is health literacy. It encompasses the understanding and ability to access, process, and apply health-related information and literacy is influenced by individual characteristics as well as social and systemic factors [4]. Particularly affected are groups with limited comprehension ability, such as individuals with lower educational backgrounds or those facing language barriers due to dementia or other cognitive impairments [5, 6]. Health literacy in socioeconomically disadvantaged populations in Germany significantly declined between 2014 and 2020 [7]. 

Consequently, physicians must communicate medical information in an understandable way so that patients can weigh up the risks and benefits. This requires adapted communication strategies and measures to promote patients’ health literacy. Furthermore, targeted measures to strengthen individual health literacy on the patient side are necessary [8]. 

For this reason, the present monocentric, prospective observational survey study aims to identify patient groups and characterize the factors that result in comprehension difficulties following a routine anaesthesia consultation.

With knowledge about the existence and needs of these groups, as well as their individual factors, physicians could tailor adjustments to their communication strategies. This could help reduce communication barriers and, consequently, poor recall or a lack of comprehension in the future. Such improvements would benefit not only patients but also the broader healthcare system.

## Methods

This was a monocentric, questionnaire-based observational survey study. The study was submitted to the responsible local ethics committee of the medical faculty of the University of Leipzig and approved by this committee (reference number 135/22). Prior to participation all patients and their anaesthesiologists gave written consent. The study was planned and carried out in accordance with all relevant guidelines and regulations and in particular with the applicable data protection laws.

All patients aged 18 years and older who attended the Pre-Anesthesia Assessment Clinic at Leipzig University Hospital for preoperative consultation were invited to participate in the study. In this anaesthesia clinic all patients prone to anaesthesia in the next days are assessed by its team. These included both complex operations and routine procedures. For the study only patients were included who presented themselves and not patients who were bedridden. The anaesthetists can inspect medical records.

After providing informed consent, participants and their respective consulting anaesthetists received an anonymous, four-part questionnaire that included multiple-choice and free-text responses. The detailed questionnaires are available in the online appendix. Data collection was performed with tablets via *evasys* software (evasys GmbH, V9.1 (2463), Lüneburg, Germany), which enables digital questionnaire distribution and response analysis. Patients were allowed to request assistance from a member of the study team to complete the questionnaire.

The questionnaire was available in German, so only German-speaking patients or those with interpreters or language mediators were able to participate.

The questionnaire was structured as follows:

- Part 1 (Pre-Interview): This section focused on personal data and patients’ self-assessments and expectations regarding the consultation and the planned procedure (questions 1.2 to 1.32).
- Part 2 (Anaesthetist Pre-Consultation Assessment): The anaesthetists were asked to evaluate the expected complexity and scope of the consultation, as well as the degree to which the procedure was particularly demanding, risky, or required detailed explanation (questions 2.2 to 2.7).
- Part 3 (Post-Consultation Anaesthetist Assessment): Following the consultation, anaesthetists were asked whether they believed the patient was able to understand the content of the discussion and process the necessary information (questions 3.2 to 3.8).
- Part 4 (Post-Consultation Patient Assessment): Patients were asked to answer content-related questions about the consultation and evaluate the quality of the discussion to assess its overall success (questions 4.2 to 4.21).

The primary aim of the study was to characterize patients with limited comprehension after the informed consent procedure; therefore, two groups were compared:

1. Group 1 “V1” (full understanding): Patients who correctly answered all comprehension-related questions (questions 4.2, 4.3, and 4.4 addressed the correct anaesthesia method, at least one of the risks of which information was provided, and possible alternative therapies discussed during the consultation based on the free text answers. These free text responses were assessed independently by 3 authors).
2. Group 2 “V0” (limited understanding): Patients who answered at least one comprehension-related question incorrectly.

**Fig. 1:**
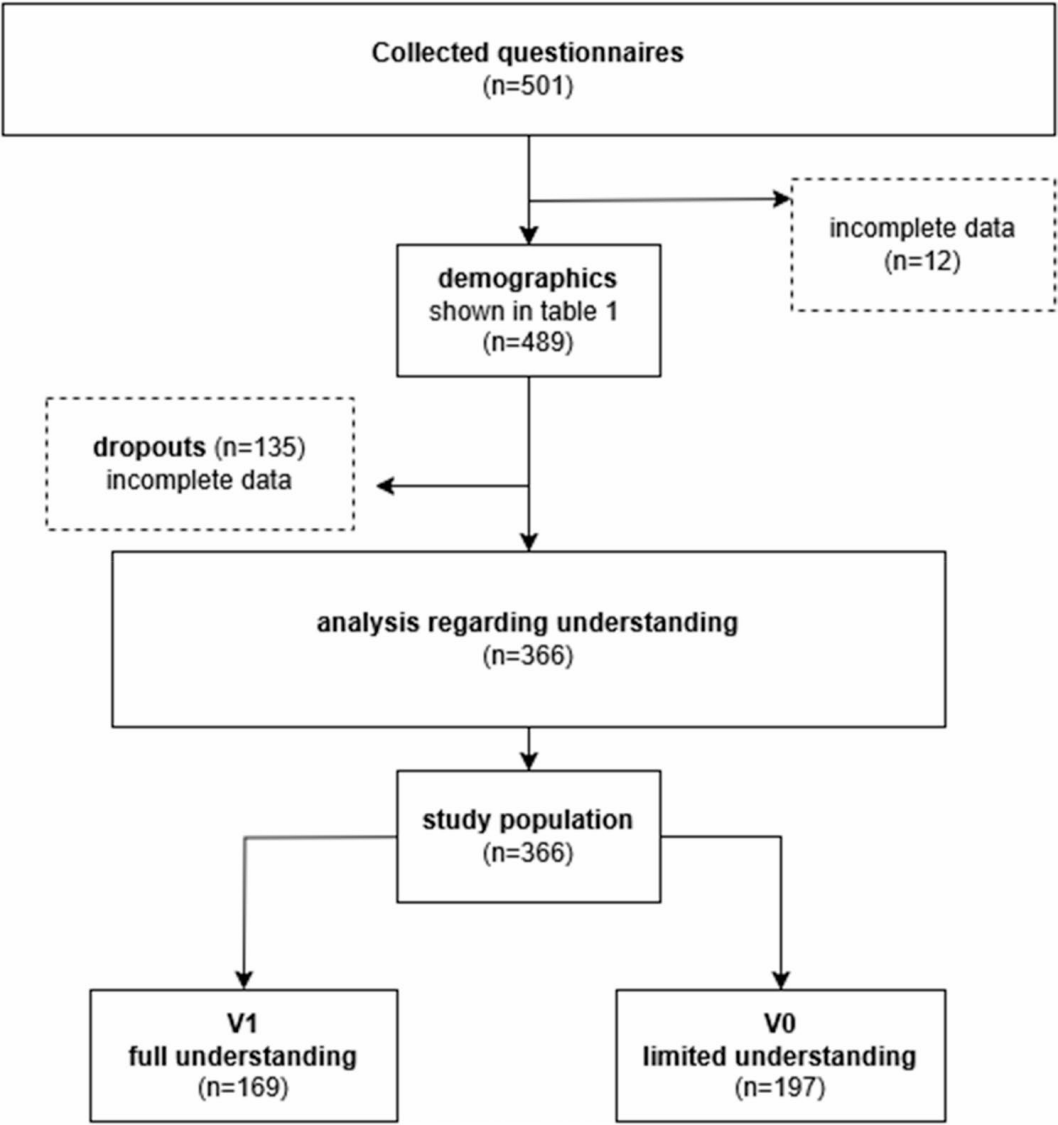
Illustration of the participants and dropouts

As this was an exploratory study, no calculation of case numbers was made. The Shapiro-Wilk test was used to assess the normal distribution. Descriptive data are presented as medians and quartiles. Comparisons between groups were conducted via Mann-Whitney U test for ordinal and continuous variables, and the chi-square (χ²) test for nominal variables. Missing values were removed from analysis. If data were incomplete that a dataset was not feasible for analysis, patients were regarded as dropouts. The manuscript was written solely by the authors. Large language models (deepL and chatGPT) were only used to refine the language and translate some passages. The flow chart (Fig. 1) was created with the freeware draw.io.

## Results

### Demographic data

From 14/06/2022 to 16/02/2024, 501 patients were included in the study. A total of 61 different anaesthetists were involved in the surveys of the 501 patients, 489 were analysed for demographic data. 12 patients were excluded in the first place due to incomplete data and a further 135 could not be used in the analysis for the comparison groups (illustrated in Fig. 1). The data were not normally distributed.

Baseline data concerning age, gender, employment status, coping with everyday life, psychiatric history, level of education, origin and language of the patients, such as the age and work experience of the anaesthetists, are listed in Table 1.

**Table 1:** Descriptive overview of the study population. Missing values are not displayed. Comparison of the two groups concerning comprehension

Patient-related questions	
Age	(*n* = 489)
Median ± std. deviation (p25; p75)	58.0 ± 17 (42.0; 67.0)
Time required to complete the patient questionnaire (min)	(*n* = 460)
Median ± std. deviation (p25; p75)	10.0 ± 6.1 (7.0; 13.0)
Gender	(*n* = 487)
Female	43.5% (*n* = 218)
Male	53.7% (*n* = 269)
Currently working	(*n* = 487)
Yes	49.7% (*n* = 249)
No (including pensioners)	47.5% (*n* = 238)
Physical competence in everyday life	(*n* = 489)
Self-sufficient	86.0% (*n* = 431)
Need for support	11.6% (*n* = 58)
Organisational competence in everyday life	(*n* = 487)
Self-sufficient	89.8% (*n* = 450)
Need for support	7.4% (*n* = 37)
Pre-existing psychiatric condition	(*n* = 486)
yes	14.8% (*n* = 74)
no	82.2% (*n* = 412)
Level of education	(*n* = 486)
no school-leaving certificate	1.6% (*n* = 8)
Secondary school certificate (9th grade)	7.0% (*n* = 35)
Secondary school certificate (10th grade)	15.2% (*n* = 76)
High school diploma (12th/13th grade)	6.4% (*n* = 32)
Completed vocational training	35.1% (*n* = 176)
Degree from a university of applied sciences	10.6% (*n* = 53)
University degree	21.2% (*n* = 106)
Parents’ and own Country of birth outside of Germany	(*n* = 501)
No	85.4% (*n* = 428)
Yes	14.6% (*n* = 73)
First language	(*n* = 488)
German	88.6% (*n* = 444)
Other	8.8% (*n* = 44)
Both parents have German as a first language	(*n* = 437)
Yes	74.9% (*n* = 375)
No	12.4% (*n* = 62)
Anaesthesia-related questions	
Duration of the anaesthesia consultation in min	(*n* = 480)
Median ± std. deviation (p25; p75)	16.0 ± 9.5 (12.0; 22.0)
Age	(*n* = 488)
Median ± std. deviation (p25; p75)	1.0(= 20–30 years) ± 0.7
20–30	56.1% (*n* = 281)
31–40	34.7% (*n* = 174)
41–50	2.8% (*n* = 14)
> 50	3.8% (*n* = 19)
Work experience in years	(*n* = 487)
Median ± std. deviation (p25; p75)	2.0 ± 4.8 (1.0; 4.0)

Table 2 shows the differences in comprehension between the two groups.

**Table 2:** Comparison between groups (full vs. limited understanding). Missing values are not displayed

	V1	V0	*p*
	full understanding	limited understanding	
	(*n* = 169)	(*n* = 197)	
Patient-related questions			
Age	(*n* = 163)	(*n* = 193)	< 0.05
Median ± std. deviation (p25; p75)	49.3 ± 17.1 (36.5; 63.0)	61.7 ± 16.0 (53.0; 74.0)	
Time required to complete the patient questionnaire (min)	(*n* = 166)	(*n* = 190)	< 0.05
Median ± std. deviation (p25; p75)	10.5 ± 6.6 (8.0; 13.0)	9.0 ± 6.1 (7.0; 12.0)	
Gender	(*n* = 161)	(*n* = 194)	< 0.05
Female	47.9% (*n* = 81)	38.6% (*n* = 76)	
Male	47.3% (*n* = 80)	59.9% (*n* = 118)	
Currently working	(*n* = 162)	(*n* = 193)	< 0.05
Yes	62.7% (*n* = 106)	37.6% (*n* = 74)	
No (including pensioners)	33.1% (*n* = 56)	60.4% (*n* = 119)	
Physical competence in everyday life	(*n* = 163)	(*n* = 195)	< 0.05
Self-sufficient	89.3% (*n* = 151)	81.7% (*n* = 161)	
Need for support	7.1% (*n* = 12)	17.3% (*n* = 34)	
Organisational competence in everyday life	(*n* = 164)	(*n* = 192)	n.s.
Self-sufficient	89.3% (*n* = 151)	88.3% (*n* = 174)	
Need for support	7.7% (*n* = 13)	9.1% (*n* = 18)	
Pre-existing psychiatric condition	(*n* = 164)	(*n* = 192)	< 0.05
Yes	18.3% (*n* = 31)	10.2% (*n* = 20)	
No	78.7% (*n* = 133)	87.3% (*n* = 172)	
Level of education	(*n* = 163)	(*n* = 192)	< 0.05
Academic	41.4% (*n* = 70)	32.5% (*n* = 64)	
Completed vocational training	27.2% (*n* = 46)	41.1% (*n* = 81)	
No qualification	27.8% (*n* = 47)	23.9% (*n* = 47)	
Parents’ and own Country of birth outside of Germany	(*n* = 165)	(*n* = 191)	n.s.
No	84.6% (*n* = 143)	85.8% (*n* = 169)	
Yes	13.0% (*n* = 22)	11.2% (*n* = 22)	
First language	(*n* = 164)	(*n* = 192)	n.s.
German	89.3% (*n* = 151)	88.8% (*n* = 175)	
Other	7.7% (*n* = 13)	8.6% (*n* = 17)	
Both parents have German as a first language	(*n* = 165)	(*n* = 192)	n.s.
Yes	86.4% (*n* = 146)	88.3% (*n* = 174)	
No	11.2% (*n* = 19)	9.1% (*n* = 18)	
Anaesthesia-related questions			
Duration of the anaesthesia consultation in min	(*n* = 161)	(*n* = 191)	n.s.
Median ± std. deviation (p25; p75)	17.0 ± 9.0 (12.0; 21.0)	16.0 ± 8.9 (12.0; 23.0)	
Age	(*n* = 161)	(*n* = 196)	
Median ± std. deviation (p25; p75)	1.0 ± 0.7	1.0 ± 0.7	n.s.
20–30	57.4% (*n* = 97)	57.9% (*n* = 114)	
31–40	30.2% (*n* = 51)	35.5% (*n* = 70)	
41–50	4.7% (*n* = 8)	2.0 (*n* = 4)	
> 50	3.0% (*n* = 5)	4.1% (*n* = 8)	
Work experience in years	(*n* = 163)	(*n* = 195)	
Median ± std. deviation (p25; p75)	2.0 ± 3.5 (1.0; 4.0)	2.0 ± 3.1 (1.0; 4.0)	n.s.

Patients whose understanding was limited were significantly older. Group V1 had a significantly greater proportion of employed patients, and academic qualifications were more common. Physical assistance needs were more than twice as high in group V0, whereas a history of self-reported psychiatric conditions was more common in group V1. Since psychiatric diagnoses were only recorded in free text and based on the patients’ own statements, answers were not evaluated in detail.”

No significant differences were found regarding factors such as the need for non-physical assistance in everyday life. Age and the professional experience of anaesthetists were not associated with patient understanding. Additionally, there were no significant differences in the proportion of patients with a foreign birthplace or who were non-German speakers.

In 94.4% of the V0 group and in 95.3% of the V1 group, the anaesthetists assumed that the patient had understood the explained procedure.

## Discussion

We identified individual factors associated with limited comprehension after preoperative anesthesia consultations. Our findings indicate associations with sociodemographic, cognitive, and situational parameters, particularly advanced age, unemployment, physical dependency, and lower educational attainment. Interestingly, patients with a history of psychiatric treatment demonstrated better comprehension.

Age emerged as a key factor, with older patients showing significantly lower levels of understanding. This aligns with previous research by Vogt et al., who found that over two-thirds of individuals aged 65 and older had difficulties processing health-related information [9]. It also can be attributed both to age-related cognitive decline, as outlined in Park et al.’s “Scaffolding Theory of Aging and Cognition” [10]and to challenges in adapting to digitalized healthcare communication. The 2020 HLS-GER2 study supports this, demonstrating lower digital health literacy scores in people aged 65 and older compared to the general population. The study also established a strong link between digital and general health literacy [11]. 

Comprehension was also significantly associated with employment status and educational attainment. Employed patients and particularly those with academic degrees demonstrated better understanding, while retired or unemployed individuals had more difficulty. This reflects previous research showing that employment and education enhance cognitive flexibility and access to health-related resources. Occupational tasks requiring multitasking and rapid information processing could enhance patients’ ability to comprehend medical concepts [12]. This underscores the potential social and cognitive benefits of employment.

Wu et al. reported that individuals with a high school or academic degrees were significantly less likely to exhibit low health literacy [13]and Jansen et al. identified education as a strong predictor of the ability to appraise and apply health information and navigating the healthcare system [14]. Additionally, higher monthly income levels are positively associated with better health literacy, highlighting the relationship between financial stability and access to health-related resources [15]. Ouyang et al. studied patients hospitalized with ischaemic stroke in China and reported that factors such as age, education level, and family income significantly influence health literacy [16]. 

Gender differences were also observed: while understanding levels were balanced in the high-comprehension group, men were overrepresented in the limited-comprehension group. This may suggest that women possess a higher degree of health literacy, potentially linked to their traditional role as caregivers in many family structures.

A study by Lee et al. (2015) demonstrated that women show greater proficiency in understanding medical information and forms. Women generally find it easier to interpret medical documents and follow instructions on medication labels. Interestingly, male participants in the same study tended to have higher educational attainment, employment rates, and income levels, yet women still exhibited better comprehension. This finding supports the hypothesis that women’s traditional caregiving roles may enhance their focus on health-related matters within families and promote stronger medical understanding [15]. 

A study by Sun et al. (2022) demonstrated also a gender-specific correlation, whereby in men, health literacy was associated with factors such as education, number of children, monthly income, duration of chronic illness and self-efficacy for chronic illness. In contrast, for women, age, education, income, duration of illness and treatment of chronic illness were found to be significant factors [17]. 

These findings underscore the notion that the comprehension of medical information is not solely determined by the level of education but also significantly influenced by the individual’s socio-economic context.

Patients requiring physical assistance in everyday life were significantly more likely to show limited comprehension, supporting existing research linking physical frailty with cognitive impairment [18, 19]. In contrast, this association was not observed in individuals needing only organizational and non-physical support. This distinction underlines the impact of physical health on cognitive and communicative capacities in medical contexts.

Interestingly, patients with a psychiatric history showed better comprehension. This might be due to their more frequent interactions with the healthcare system, leading to improved familiarity with medical terminology and procedures. Schomerus et al. observed increased recognition of mental illness and greater likelihood of seeking help in individuals with psychiatric experience [20]. As previously outlined, Lee et al. also identified depression as a gender-independent predictor of health literacy [15]. Furthermore, evidence suggests that there may be a distinction in the various psychiatric disorders. According to Sato et al., health literacy levels are greater in individuals diagnosed with anxiety disorders than in those with schizophrenia or affective disorders [21]. 

In the absence of a universally accepted definition of a migrant background, we inquired about factors that might have influenced the comprehension of an education interview, whether by origin, migration background or the patient’s first language.

The study revealed no statistically significant differences between the groups, a finding that aligns with prior observations by Schaeffer et al. However, these earlier studies focused on digital health literacy, but the multivariate analysis indicated a strong relationship between digital and general health literacy. Notably, individuals with a parental history of migration presented higher levels of digital health literacy compared to the general population [11]. 

The lack of significant difference may also be attributed to the low diversity of the study population, as the study was conducted in Saxony, Eastern Germany, where the history of German separation still results in a lower proportion of non-native speakers and lower migration rates [22]. 

Counter to expectations, no significant differences were observed in the duration of the consultation between the two groups, suggesting that clarity and quality of communication matter more than time spent. Vogele et al. demonstrated that satisfaction increases with better communication, with video and tablet-based aids viewed positively by patients [23]. Hoffmann et al. found that tailored discharge letters significantly improved patients’ understanding and application of medical instructions [24]. These findings could indicate that the way in which content is communicated (for the purpose of visualization, repetition, summaries, etc.) plays a more decisive role than the pure duration of doctor-patient consultations.

While our study focused on immediate comprehension during anesthesia consultations, the concept of health literacy—defined as the ability to access, understand, evaluate, and apply health-related information [5]—remains relevant.

As a key factor in navigating complex healthcare systems, it enables individuals to interpret medical information in context, make informed decisions, and actively participate in their own and their community’s health, while limited health literacy can hinder these processes, especially in stressful settings like preoperative care [7]. 

The “European Health Literacy Survey (HLS-EU),” conducted across eight European countries, revealed that, on average, one in two people has limited health literacy. The survey also established an association with social determinants: limited health literacy is more common among individuals with lower socioeconomic status, lower education, older age, and impaired cognitive function [5, 6]. Other studies have confirmed a high prevalence of low health literacy (34–59%). [25] There is also a logical connection between limited health literacy and the occurrence of specific diseases. For example, a correlation has been observed between low health literacy and higher rates of heart failure [6]. 

The informed consent process in a Pre-Anesthesia Assessment clinic may also be negatively influenced by limited health literacy, as a variety of personal and external factors affect how complex medical information is received and understood.

Informed consent discussions represent a critical interface where the relevance of health literacy to individual healthcare becomes evident. Limited health literacy can prevent patients from fully understanding or applying key information, which significantly impairs their ability to make informed decisions about their own health.

Overall, our findings underscore the importance of identifying vulnerable patient groups and tailoring communication strategies accordingly. Targeted approaches—such as simplified language, visual tools, and digital aids—may support better comprehension. Improving communication not only benefits individual patients but also strengthens the broader healthcare system by reducing misunderstandings and improving adherence.

### Limitations

- A key limitation of the study is its monocentric design, which was conducted in a single region of Germany. Additionally, its focus on the outpatient sector precludes the inclusion of bedridden patients. Consequently, the study is unable to achieve universality, and its conclusions must be considered within the context of its specific methodological framework.
- The variability in the age and experience of anaesthetists was insufficiently captured, as junior physicians conducted most consultations.
- To secure anonymity, also no identifier was stored for the anaesthetist. Consequently, we can not exclude that some anaesthetists are more effective at providing information than others.
- The use of a standardized questionnaire in German excluded non-German-speaking patients.
- The questions regarding birthplace other than Germany and first language other than German are likely to have lacked sufficient discriminatory power to capture a language barrier between the patients and the German language. A subjective assessment of patients’ understanding of German would have been a more useful tool.

## Conclusion

Advanced age, lack of employment (including retired patients), physical dependency, and lower educational attainment were significantly associated with limited comprehension during anaesthesia consultations. Interestingly, patients with a self-reported history of psychiatric treatment presented fewer comprehension gaps. Future research should focus on developing and implementing tailored interventions to improve individual understanding of medical information and to enhance health literacy across the population.

## Supplementary Information

Supplementary Material 1.

## Data Availability

Due to German data protection laws and following the EC approval we regret not to be allowed to publish pseudonymized data. The datasets generated and analyzed during the current study are available from the corresponding author on reasonable request.

## Abbreviations

HLS-EU: European Health Literacy Survey
HLS-GER: German Health Literacy Survey
V1: Group with full understanding
V0: Group with limited understanding
χ²: Chi-square test
DGK: Digital Health Literacy Score
HL-DIGI-HI: Health Literacy Digital Health Indicator
AZ: Reference number (ethics committee number)
