# Supplementary material for: Limitations of informed consent in routine clinical practice - An observational survey study to characterize patient populations with limited understanding of the routine informed consent process in an anaesthesia clinic
Source: BMC Anesthesiol. 2025 Jul 30;25:378. doi: 10.1186/s12871-025-03275-9 (PMC12312230; doi:10.1186/s12871-025-03275-9)
Supplement: Supplementary file 1 — Supplementary Material 1. [file 12871_2025_3275_MOESM1_ESM.docx]

Translated questionnaire (from German to English)

**1. PATIENT ANTE (before the Anaesthesia consultation)**

1.1 Please note the current time (free text field)

1.2 Please tell us your age (years) (free text field)

1.3 Please tell us your gender (single choice from defined options: male/female/divers)

1.4 Do you currently practice a profession? (single choice from defined options: yes/no)

1.5 If yes: What is your profession? (free text field)

1.6 How do you do everyday things (e.g. shopping, personal hygiene)? (single choice from defined options: self-sufficient/need support from friends, family, partner, flatmate,../ I receive support by a nursing service/ I live in a care facility/ Other (please specify free text field))

1.7 Free text field: Other support for everyday things

1.8 Do you need support with organisational tasks (e.g. account management, handling money)? (single choice from defined options: No/ I am supported by friends, family / I have an legal carer / Other (please specify/free text field)

1.9 Free text field: Other Support with organisational tasks

1.10 Have you ever been treated by a psychiatrist or in a psychiatric clinic? (single choice from defined options: yes/no)

1.11 If yes: What diagnosis was made? (free text field)

1.12 What is your highest qualification from an educational institution? (single choice from defined options: I have no School-leaving certificate / Secondary school certificate (9^th^ grade) Secondary school certificate (10^th^ grade) / A-levels Completed / completed Vocational training (apprenticeship) / Degree from a university of applied sciences / University studies)

1.13 In which country were you born? (free text field)

1.14 In which country were your parents born? (free text field)

1.15 What was the first language you spoke? (free text field)

1.16 What is your father's mother tongue? (free text field)

1.17 What is your mother's mother tongue? (free text field)

**Why are you here in the anaesthesia outpatient clinic? Please rate the extent to which the statement applies:** (the following questions 1.18 -2.18 could be answered with a Likert scale with four possible options (single choice) from strongly agree - strongly disagree)

1.18 I have been sent here and I don't know exactly what is going to happen.

1.19 The anaesthetic consultation is a formality that I want to get over with quickly.

1.20 The surgeon says I need an operation to make me feel better. I can't do it without an anaesthetic.

1.21 Important questions are clarified here to ensure a safe operation.

1.22 I would like to be informed in detail about the procedure and possible complications so that I know what I am letting myself in for.

1.23 I don't really have any questions and want to get this over with quickly.

1.24 I have a lot of questions about anaesthesia and would like to find out more here.

1.25 I am afraid of anaesthesia and hope that my fears will be allayed.

1.26 I am due to have an operation because of my illness. I would like to have the operation so that I feel better.

1.27 I am not interested in what happens during an anaesthetic. The main thing is that I wake up afterwards.

2.18 I think I know pretty well what happens during an anaesthetic and have no questions.

**What are your most important concerns or questions for this conversation? You can name 3 aspects.**

Concern/question 1 (free text field)

Concern/question 2 (free text field)

Concern/question 3 (free text field)

1.32 Where did you get information about what is going to happen here? (single choice from defined options: I have no information about this / I was treated by my surgeon / I already have own experience with operations and anaesthesia / I have informed myself beforehand through publicly accessible sources (e.g. Internet) /I have informed myself in private environment / I have learnt through my medical profession)

1.33 Please note the current time (free text field)

**2. ANESTHESIOLOGIST ANTE (before the Anaesthesia consultation)**

2.1 Please note the current time (free text field)

2.2 How old are you? (single choice from defined options: 20-30 years / 31-40 years / 41-50 years / >50 years)

2.3 How many years of professional experience do you have as a doctor? (free text field)

**Assessment of the upcoming conversation:** (the following questions 2.4-2.8 could be answered with a Likert scale with four possible options (single choice) from strongly agree - strongly disagree)

2.4 Routine procedure without particular risks

2.5 Critical medical questions that are of particular importance for the course of the procedure are to be clarified. I expect the consultation to be of particular importance to the patient and will need to take time to answer questions.

2.6 I must discuss relevant questions about the procedure (e.g. handling of medication) with the patient.

2.7 I must discuss relevant treatment alternatives (e.g. regional anaesthesia) with the patient.

2.8 Please note the current time (free text field)

**3. ANESTHESIOLOGIST POST (after the Anaesthesia consultation)**

3.1 Please note the current time (free text field)

(the following questions 3.2-3.8 could be answered with a Likert scale with four possible options (single choice) from strongly agree - strongly disagree)

3.2 I was able to take time for all the patient's questions.

3.3 I have done justice to the patient.

3.4 The patient understood the procedure well.

3.5 The patient was interested in the explanatory discussion.

3.6 The patient just wanted to get the consultation over with quickly.

3.7 The patient had difficulty following me.

3.8 The patient was well informed and only needed a little additional information.

3.7 Please note the current time (free text field)

**4. PATIENT POST (after the Anaesthesia consultation)**

**Please answer the following questions briefly in your own words (free text).**

4.1 Please note the current time (free text field)

4.2 Which anaesthesia and / or pain procedure should be performed? (free text field)

4.3 Are there other options for anaesthesia / pain management? (free text field)

4.4 What are the risks associated with the proposed procedure? (free text field)

4.5 What would happen if you do not agree? (free text field)

Please rate the following statements: (the following questions 4.6- 4.21 could be answered with a Likert scale with four possible options (single choice) from strongly agree - strongly disagree)

4.6 My expectations of this consultation have been met.

4.7 My questions have been answered.

4.8 I have understood the planned procedure.

4.9 I know what general risks I have to bear.

4.10 There are risks that specifically affect me. I now know these.

4.11 I don't care about my personal risks. I have no choice anyway.

4.12 Alternatives to this procedure have been discussed with me.

4.13 I have understood which anaesthetic procedure will be used.

4.14 My medical history was taken.

4.15 I had the opportunity to get rid of my fears.

4.16 I felt understood.

4.17 There was enough time for me to ask all my questions.

4.18 The anaesthetist left room for questions.

4.19 I had the feeling that I should sign quickly because many patients were still waiting.

4.20 I think there is still enough time before the operation. So I can ask other people for advice.

4.21 I could have saved myself the conversation. The operation has to be carried out anyway.

4.22 Please note the current time (free text field)
